# Making Sense of Dementia: Older Adults’ Subjective Representations of Dementia and Alzheimer’s Disease

**DOI:** 10.1093/geronb/gbae056

**Published:** 2024-04-04

**Authors:** Jaroslava Hasmanová Marhánková, Michaela Honelová

**Affiliations:** Institute of Sociological Studies, Faculty of Social Science, Charles University, Prague, Czech Republic; Institute of Sociological Studies, Faculty of Social Science, Charles University, Prague, Czech Republic; (Social Sciences Section)

**Keywords:** Dementia worry, Discourse, Fourth age, Language

## Abstract

**Objectives:**

This research explores how the representations and meanings of living with dementia are constructed by older adults.

**Methods:**

Focus groups (*N* = 19) and in-depth interviews (*N* = 29) were conducted with older adults aged 65+ living in the Czech Republic, representing different levels of personal familiarity with care for an individual experiencing dementia.

**Results:**

We identified 2 different discourses: (1) *Tragedy discourse* with two distinctive repertoires “dementia as a thief of personality” and “dementia as a thief of humanity.” Within such discourse, dementia transcends mere medical terminology, serving as a symbolic representation of existential anxieties linked to aging and the perceived loss of control. (2) The discourse of *Dementia as a specific way in which people approach the world* was articulated mainly by caregivers, providing them with a coping mechanism and a means to reconstruct the agency of the person experiencing dementia. In older adults’ representations, references to suffering among family members emerged as a primary association with dementia. Dementia was portrayed as “contagious” in its effect on the family members who were, in a sense, depicted as the primary sufferers of the disease.

**Discussion:**

Dementia often serves as a symbolic tool for older adults to articulate concerns about advanced old age, extending beyond its clinical definition to convey deep-seated fears associated with aging. The experience of people surrounding those diagnosed with dementia and the permeability of the impacts of this disease between bodies represented crucial frameworks for conceptualizing dementia in the narratives of older adults.

Dementia (see Author Note 1) figures prominently in the cultural imaginary of the fourth age as the marker of the fearful condition of “frail” old age (Higgs & Gilleard, 2014). Although systematic efforts have been made to disentangle dementia from the “normal” process of growing old, its social representations continue to blur the line between the medical condition and the fears associated with advanced old age (Marhánková, 2023). As indicated by Lock (2014), Alzheimer’s disease (AD) embodies all fears about growing old.

Dementia represents one of the most frequent fears people have about aging (Kessler et al., 2012) while remaining a taboo topic often avoided in personal conversations and even healthcare consultations (Andrews et al., 2017), making it hard to verbalize what dementia means. Existing research has focused mainly on the representation of dementia/AD articulated by persons living with (mostly early-stage) dementia (Beard & Neary, 2013; Langdon et al., 2007; Lingler et al., 2006) or their family members (Golden et al., 2012). In our research, we focus on the perspective of older adults not diagnosed with dementia to investigate how they understand it and what language they use to describe and capture the symptoms associated with such a condition. We aim to reconstruct how dementia is narrated in the lay understanding of those who may be positioned as being at risk of this condition due to their higher chronological age. This study explores the tools, metaphors, and sentiments that older adults mobilize in their subjective understanding of what dementia represents and in defining the essential features of such a condition.

## Social Representations of Dementia

The information that dementia cannot be considered a normal part of aging currently guides all public documents on dementia. However, this effort to distinguish dementia from the image of a “normal” process of aging contrasts with the remaining specific position of dementia in the social representation of older age. As indicated by Zeilig (2014, p. 262), dementia and AD remain strongly value-laden terms whose substance is communicated through metaphors and cultural stories that embody the combination of anxiety about old age and mental illness. The figure of an older person living with dementia personifies the negative image of growing old badly (Latimer, 2018). Dementia/AD is framed using specific language referring to catastrophizing images of a demographic time bomb (Peel, 2014), epidemic (Low & Purwaningrum, 2020), or war (George et al., 2016; Lane et al., 2013). AD/dementia is depicted as a major threat to what created a personhood, evoking the images of “loss of self” and social death (Behuniak, 2011; George, 2010; Sweeting & Gilhooly, 1997).

The emphasis on the positive discourse of “living well with dementia” could be considered an attempt to remedy the negative representation of dementia/AD. Inspired especially by the person-centered approach outlined by Kitwood (1997), these discourses frame dementia with an emphasis on dignity and meaningful experiences of both those who live with dementia and their close ones. However, as pointed out by McParland et al. (2017), although the “living well” discourse represents a polarized counterimage to the “tragedy discourse,” it predominantly focuses on the emphasis of “being the same,” on living well and having value despite dementia, rather than rethinking the very definition of normality and value that problematize the social responses to dementia/AD (McParland et al., 2017, p. 266).

Significant attention has already been paid to the anxieties that dementia evokes among older adults (Kessler et al., 2012). Dementia worry might be increased by exposure to negative stereotypes of older age, especially among those who self-identify as older adults (Molden & Maxfield, 2017), suggesting that dementia worry represents a combination of anxieties regarding aging and health anxiety (Kessler et al., 2012) and can be, to a certain extent, attributed to internalized ageism (Yun & Maxfield, 2020). Another distinctive stream of research explores the levels of dementia literacy in terms of knowledge, understanding, and awareness that individuals possess about dementia and related conditions, including knowledge of symptoms, risk factors, and available treatments (i.e., Anderson et al., 2009; Low & Anstey, 2009; Nagel et al., 2021). Older adults have different levels of dementia literacy, and the knowledge about dementia is closely related to cultural values and attitudes (e.g., Cipriani & Borin, 2015; Sun et al., 2015).

How people talk about dementia/AD shapes our collective understanding, perceptions, and attitudes toward such conditions. The meanings attributed to certain illnesses affect stigma associated with the health condition that may further affect health strategies, such as attitudes toward screening (Casado et al., 2018; Marhánková, 2023) or healthcare professionals’ proactive approach to diagnosing dementia (Cahill et al., 2008). A deeper analysis of how individuals construct and understand what dementia means is essential for creating more dementia-respecting communities. It helps us understand the mechanism contributing to the stigmatization of individuals living with dementia and the anxieties such a condition evokes. Understanding older adults’ perceptions of dementia/AD may be particularly important in the context of the role dementia plays in the representation of (advanced) old age. As the risk of developing dementia increases with age, understanding how older adults communicate about and perceive dementia provides valuable insights into the challenges they may face as they age. As proposed by stereotype embodiment theory (Levy, 2009), negative meanings attributed to older age may influence numerous cognitive and physical outcomes. Understanding how older adults talk about dementia is vital not only for improving the support and care for individuals living with dementia but also for comprehending the broader implications of age-related stereotypes on older adults themselves.

## Method

### Study Design and Participants

The data presented in this study represent a subset of a larger project entitled *Social Life of Dementia*, which explores the social representations of dementia, both in the media and social policy discourses in the Czech Republic and in the subjective attitudes of older adults (people aged 65+ years).

Two data production techniques were employed in our research. We conducted 29 in-depth interviews with older adults (aged 65+ years) living in the Czech Republic. We interviewed participants living independently in their households who had not been diagnosed with any form of cognitive impairment. All study participants were recruited using social networks, contacts provided by organizations that offer leisure-time activities for older adults, and snowball sampling. We struggled to capture as diverse experiences as possible (our participants differed in their education and former occupations; some lived in large cities, while others lived in rural areas). However, we are aware that our sample may not capture all of the heterogeneity of the experience of aging. Our participants were mainly older adults with a higher level of social engagement who framed their retirement life as a positive experience. Women were slightly overrepresented in our sample (*N* = 20). During the sampling, we focused on representing various age groups (ages 65–74 years: *N* = 16, ages 75+ years: *N* = 13) and different levels of personal familiarity with care for someone experiencing dementia. Twelve participants stated that they did not know someone from their immediate surroundings who was diagnosed with dementia/AD. Eleven of the participants were informal carers for a family member (parent, parents-in-law, spouse) living with dementia/AD at some point in their lives. The remaining six participants mentioned knowing a person who was diagnosed with dementia/AD.

Individual interviews were supplemented by four focus groups with older adults (*N* = 19). In particular, there were two groups of women, the first aged 65–75 and the second aged 76+. The other two groups were again mixed (men and women) in the same age categories. Two focus groups were conducted in a residential facility for older adults who did not require assistance in their daily activities. The two remaining focus groups were organized with clients of the center for older adults offering leisure-time activities (e.g., computer courses, public lectures, and sports activities). Women were overrepresented in our sample (*N* = 16), a fact that reflected the structure of the clients of the facility/center that assisted in focus group organization. The participants were recruited with the assistance of employees at the facility. In addition to chronological age, the inclusion criteria were set for participants without a personal experience of caring for a person living with dementia/AD. Due to the topic’s sensitivity, we did not plan to involve people who were or still are primary caregivers in the focus interviews. However, four of the participants who were former caregivers expressed an explicit desire to participate in the discussion and share their experiences. All participants were informed in advance regarding the topic of the discussion and the potential sensitivity of the topic. We paid close attention to ensuring that only adults who felt comfortable sharing their attitudes and experiences participated in the study. We assured all participants that this is a safe environment and that they do not have to answer any questions that would make them uncomfortable.

### Data Collection and Analysis

The interviews and focus groups typically lasted between 50 and 90 min and were recorded and transcribed with the participants’ consent. The interview guide was structured to cover the following aspects: (1) daily life after retirement and attitudes toward aging, (2) future expectations, (3) lay understanding of dementia/AD, and (4) concerns related to being at risk of dementia, attitudes, and experiences regarding possible screenings and medical consultations for AD. The analysis presented in this paper focuses on the third domain, which addresses the meanings the participants associated with dementia. Two key questions were employed to stimulate the answers: “What comes to your mind when someone says dementia or Alzheimer’s disease?” and “How would you explain what dementia or Alzheimer’s disease is to someone who has no information about it?” During the first in-depth interviews, we separated the questions regarding dementia and AD, asking for specific answers regarding these conditions separately, as well as for the participants’ reflections on whether they perceived some difference in these terms. However, during these interviews, the participants expressed confusion. Therefore, we decided not to strictly distinguish between these terms (although we are aware that they cannot be considered synonymous).

The verbatim transcriptions of the interviews were analyzed using NVivo software. In the first step, we followed the principles of thematic analysis (Ezzy, 2002) to identify key features and rhetorical tools that the participants employed to describe dementia/AD. In the case of the individual interviews, we focused on analyzing the data in the context of the participants’ personal biographies (especially with respect to the personal history of care). Our analysis was guided by the following research questions: What kind of defining features do participants associate with dementia/AD? What kinds of rhetorical devices and descriptions are used to communicate what dementia means?

### Context

In 2022, almost 123,000 people in the Czech Republic were diagnosed with some form of dementia. Regarding population, roughly one in 85 people in the Czech Republic live with this diagnosis. Similar to other European countries, we can observe a relatively steep increase in the prevalence of dementia. The number of people living with AD in the Czech Republic has multiplied 2.5 times in the last 10 years (Ústav zdravotnických informací a statistiky ČR, 2023). These figures are only indicative. Available studies suggest that underdiagnosis is occurring in the Czech Republic. There are no studies that map health literacy directly concerning dementia and AD. However, fragmentary research suggests that compared to other European countries, caregivers are more likely to interpret symptoms of dementia as problems with aging rather than health conditions (Woods et al. 2019). Much of the care provided to people living with dementia is provided by informal carers (rough estimates suggest that around 70% of care provided takes place in the family). Two to three family members often care for a person with dementia (Mátl et al. 2016).

Like other post-socialist states, the Czech Republic has had a complicated history of transitioning from a relatively early process of institutionalizing eldercare to emphasizing familialism. The onset of the communist regime in Czechoslovakia in the 1950s was characterized by an accent on the institutional form of care and a conscious effort to free the female labor force from the demanding care responsibilities at home. Under communism, Law No. 174/1948 was passed, bringing about significant changes in social services. The state became the sole legislated provider of all social services. Eldercare politics were oriented in this direction to liberate women from the demanding care responsibilities at home and to free the female labor force. However, in the 1960s and 1970s, these efforts shifted toward familialism. The return to an emphasis on the family as the primary and best place to care for older adults was driven not so much by a change in social policy priorities as by the lack of capacity of institutional facilities and their generally poor quality. The trend toward familialism was further strengthened after the fall of the communist regime in the 1990s (Souralová and Šlesingerová 2017).

Czech society has relatively strong normative expectations regarding the role of the family in caring for older family members. Assisted living facilities and nursing homes are generally considered the last possible choice (Souralová and Šlesingerová 2017). However, despite the prevailing preference for family caregiving, the demand for institutionalized care is still growing. According to available statistics, in 2021, there were 526 senior residential care homes in the Czech Republic with a capacity of just under 36,000 beds. In the same year, there were 376 homes with special arrangements (Czech Statistical Office, 2022).

## Results

The participants employed two approaches to communicate the description of dementia. The caregivers typically *portrayed the disease through symptoms* that they observed in their loved ones, such as memory loss, spatial disorientation, difficulty performing basic activities, changes in mood or behavior, or failure to recognize objects. In addition to the symptoms, the caregivers used medical terminology to explain how dementia begins or progresses.

I guess it’s the loss of mental functions, cognitive functions, for us, the worst is the spatial orientation. But it goes one with the other. He can’t find the toilet, and he goes in there, and he doesn’t know what to do. So, it’s not just space; it’s more than that. (Mrs. Sylva, age 77, caregiving experience)

In contrast, the noncaregiving respondents *described the disease more through its effects*. These were explicitly associated with loss of agency, dependence on care, caregiving burden, or tragedy for the family.

An unhappy family, because the person does nothing and the people around him suffer, and the man doesn’t think. (Mrs. Olga, age 83)

The prominent feature that emerged from the participants’ descriptions of dementia was *personalization*. Individuals frequently personalized their accounts, drawing upon either their own caregiving experiences or the narratives they encountered in popular culture to describe what dementia meant to them. Several respondent-caregivers portrayed the disease with the specific person they care for: “now, my husband,” “now my wife,” “my mother.” They often used language that did not distinguish between the person and the illness (see also Hall & Sikes, 2017). Those without personal caregiving experience often employed the descriptions of their neighbors or acquaintances or described dementia by referring to films that deal with dementia or Alzheimer’s.

A different typology of the meanings of the disease was crystallized in the analysis of the interviews. A distinction between a strictly negative discourse of dementia as an unparallel horrific condition and a discourse focusing more on the understating of the experience of living with dementia was identified in the narratives of older adults. However, these approaches were not strictly dichotomized, as suggested by the distinction between discourses of tragedy and aging well with dementia (McParland et al., 2017). Rather than opposing discourses, they represented different repertoires of articulating what constitutes dementia as a terrifying and tragic condition. Nevertheless, these discourses differ significantly in terms of both the meanings attributed to dementia and the role of these discourses in coping with the anxiety associated with dementia/AD.

### Tragedy Discourse: Dementia as a Thief of Personality Versus Dementia as a Thief of Humanity

We identified two specific repertoires of the tragedy discourse—dementia as a thief of personality and dementia as a thief of humanity—both of which focused on describing dementia/AD in terms of social death (Sweeting & Gilhooly, 1997). The discourse of *dementia as a thief of personality* was prevalent among older adults with personal experience of caring for the family member(s) living with dementia. Caregivers described dementia/AD and its progress as the gradual loss or erosion of personality concerning the loved one they cared for.

It’s just irreversible. It robs a person of his/her personality like, well, because my dad, he used to forget, he used to forget, and then his memory got stuck by the time he was 35. (Mr. Simona, age 72, caregiving experience)

Dementia/AD in these descriptions represented an erosion of the self, indicating the centrality of the memories for constructing individual uniqueness and personality (Strikwerda-Brown et al., 2019). Dementia/AD was constructed through a reference to a particular symptom (memory loss) that distinguished the specificity of such a condition and its consequences. This construction contrasted with the second repertoire of tragedy discourse that was prevalent among older adults without caregiving experience. In these descriptions, dementia/AD was similarly constructed in terms of social death. However, noncaregivers discussed the situation with more impersonal metaphors and referred to an overall loss of humanity. Paradoxically, the participants using the repertoire of *dementia as a thief of humanity* usually avoided reference to dementia/AD as a specific disease. Dementia was communicated through a more general description of the imaginary of the fourth age (Gilleard & Higgs, 2010). The participants in these cases often avoided reference to symptoms or even the reference to dementia as such. Their responses focused on describing fearful images of dependency, loss of autonomy, and dignity. In these narratives, dementia served primarily as an implicit framework for constructing the boundary distinguishing “good old age” from the life condition that is not worth living. By answering the questions regarding the meanings of dementia, the participants primarily articulated their concerns regarding the “dreaded” old age.

Well, because the person is no longer a person, it is the body that lies, which the nursing staff feed or not. (Mrs. Lenka, age 76)

### Dementia as a Specific Way in Which People Approach the World

The second discourse was identified only among participants who had experienced caring for relatives living with dementia. This description of dementia focused on its conceptualization as a disease that primarily changes how an individual interprets the world around themselves and whose behavior is affected by this change in perspective. Such descriptions did not put aside the reference to dementia as a serious and horrifying condition; however, they simultaneously enabled a positive reframing of the behavior associated with dementia. Dementia was described primarily as a different way of approaching the world that has a different logic because the individual cannot follow the previous patterns. For example, Mr. Jaromír, who was providing care to his mother-in-law diagnosed with AD at the time of the interview, responded to our question (How would you describe the disease to someone who has no information about it?) as follows:

Now, if she’s (his mother-in-law) looking for something and we ask what it is, she fabricates some answer because she’s shy and doesn’t want to say that she can’t find the right door. But she is very aware of that, so she’s basically an intelligent person with no memory. So, it’s kind of hard to get used to, and it’s kind of scary, okay. It’s annoying. And you have to take it easy on such a person. I know that I avoided your question. I keep telling you not what Alzheimer’s is but how one should approach it. I can’t describe otherwise. (Mr. Jaromír, age 69, caregiving experience)

The emphasis on dementia as a disease, together with that on the need to interpret and understand the behavior of people living with dementia in the context of the altered mode of perception, enabled the participants to reconstruct a meaningful relationship with the close one living with dementia. These narratives often focused on the description of seemingly irrational behavior, which, however, may be guided by a different kind of rationality.

They don’t make things up; they perceive them that way. I have a good example. One lady at the nursing home kept on yelling. And I thought to myself, Jesus, why is she still screaming? And as she was lying in bed, there was a picture of a dog, a bulldog, and it looked threatening from below, so when she saw the photo below, she was afraid of it and screamed. And they didn’t know why she was screaming for a long time. Then, they took down the painting, and there was no screaming anymore. (Mrs. Marcela, age 66, caregiving experience)

Such a perspective enabled to give the sense of dementia as a condition that alters but does not destroy self and agency. Compared to the *tragedy discourse*, this conceptualization was distinguished by two particular features. First, it served as an important form of coping mechanism for carers. The focus on recognizing that the behavior exhibited by individuals living with dementia/AD may have a different kind of rationality and is not intentional, alleviate the feelings of frustration, anger, or blame. While the dynamics may be different from before, the emphasis on recognizing the meaningful background of a seemingly irrational behavior helped caregivers maintain a sense of connection and search for meanings and patterns within the context of the person’s altered perception. This form of conceptualization of dementia was identified only among a few carers. In all of these cases, the participants expressed a less negative attitude toward care. These participants stressed that the understanding of dementia/AD as primarily a medical condition provided a sense of guidelines to navigate behavior and attitudes to their close ones. Mrs. Marcela framed her depiction of dementia using validation theory, offering a counterdiscourse to the conceptualization of dementia/AD as the “thief of personality.” Her interpretation of her father-in-law’s seemingly irrational behavior referred to his unique life experience that may not be lost in dementia but is only communicated differently.

I agree with the author who invented the validation that those with dementia are solving the problems they didn’t have solved in middle age. I’ll give you maybe an example: the nurses in nursing home gave my father-in-law, who had dementia, a bed bars so he would not fall during the night. I came in the morning and I’d ask him how he was doing, and he’d start screaming: “call my lawyer right now”. And I’m like, what’s a lawyer good for now? And then I remember that he was a political prisoner and he was in prison for an awful long time and I think that the bed reminded him of this experience. (Mrs. Marcela, age 66, caregiving experience)

All participants who constructed this representation of dementia explicitly highlighted that their perception of dementia changed over time and that their current perspective was influenced by the professional discourses communicated, especially, by the organization and self-help groups for carers. For example, Mrs. Marcela highlighted her previous medical experiences and the influence of the validation theory she studied during her professional career. Mr. Vladimír pointed out the influence of his involvement in Alzheimer’s cafes, stressing that he had to “learn” how to interpret and approach his wife’s behavior.

### Body Permeability: The Role of Family and Others in the Representations of Dementia

The depiction of dementia was firmly embedded in the social effects of the health condition. Surprisingly, while depicting the symptoms and course of dementia, the participants more often focused not on the behavior and condition of the individual experiencing dementia but on the family and close ones of this imagined person. There was a common emphasis on dementia as a condition that brought great suffering. However, this suffering was not primarily associated with the person diagnosed with dementia. In contrast, there was a reappearing metaphor of dementia as a form of “anesthesia” that numbs the pain for those living with such a condition while strengthening the suffering in the case of their loved ones.

They (people living with dementia) don’t even realize it, right? And that’s why they’re happy; they’re happy in a way. We feel sorry for them, but they live in their little world and are happy. So, I think people around them are suffering, they’re not suffering. (Mr. Jan, age 78)

There was a contrast between the burden of care associated with dementia and the effect of liberation that comes with the gradual loss of awareness that one is experiencing some form of health limitation. Dementia was depicted as a disease that is primarily felt by others and not those who may experience it. One of the fears that the participants often articulated was the feeling that they might already be expressing some symptoms of dementia. However, unlike those around them, they would not be able to recognize them, as illustrated by the following excerpt from the focus group discussion:

What comes first to your mind when someone says “Alzheimer’s disease”?

Eva: The end.

Oli: Huge problems for the family and the surrounding, for the people close to them.

Ester: I’m afraid that there is even no self-reflection at some point. That the person does not even know that he suffers from the disease, and that terrifies me. The situation when you don’t even know about it.

Eva: But the others know.

Dementia/AD were depicted as conditions primarily visible to others. Simultaneously, the family and close ones were positioned at the center of the effect of dementia. The boundary between the bodies affected by dementia was blurred. AD was depicted as “contagious” in its effect on the family members who were, in a sense, depicted as the primary sufferers of the disease.

When you say Alzheimer’s disease or dementia, what’s the first thing that comes to mind?

An unhappy family because the person does nothing and the people around him suffer, and the man doesn’t know about it. That’s the only thing I’m afraid of … When you see it like that or hear around how those families have it at home, that’s what it is. It’s a mess. (Mr. Oldřich, age 68)

During the interviews, the participants articulated their anxieties regarding dementia (and being at risk of developing dementia in the future). The effect of the diagnosis on the family and the burden of care associated with such a condition represented a critical framework for articulating those fears. Dementia was constructed as a health condition that disrupts social relationships. Any severe illness affects the environment and the lives of the close ones of the person experiencing such a condition. However, we would argue that in the case of dementia and AD, the experience of people surrounding those diagnosed with AD and the permeability of the impacts of this disease between bodies represent crucial frameworks for how AD is conceptualized in the narratives of older adults. In the case of AD/dementia, the bodies were constructed as permeable, creating a representation of dementia as a condition that trespasses the boundaries of individual bodies. The “contagiousness” of the effect of dementia and the transfer of suffering in the participants’ descriptions of dementia indicate the prominent role of social relationships in the subjective representations of dementia. The central aspect of the suffering of the family and close ones points out the prevalence of the tragedy discourse (McParland et al., 2017) in the subjective representation of dementia among older adults. The tragedy in the description of most of the interviewed older adults was related to the feelings and situations of others, further exacerbating the image of dementia as a condition that strips the individual of their feelings and personality. The images of what dementia and AD mean were communicated not through the descriptions of the health conditions or symptoms, which would place the individuals experiencing dementia/AD at the core of understanding these conditions. Instead, the focus predominantly lies on the impact of these conditions on others, thereby inadvertently overshadowing and marginalizing the lived experiences of individuals living with dementia themselves.

## Discussion

Our study shows that older adults conceptualize dementia not merely through the lens of its symptoms or biomedical definitions but rather through emotional responses and personal stories. The older adults interviewed in our research most often communicated their subjective understanding of dementia through strong emotional responses, such as anxiety and personal fears. These emotional reactions highlight the profoundly personal and intimate nature of dementia’s impact on individuals and families.

Dementia, in the older adults’ descriptions, emerges not just as a medical condition but as a symbolic representation of the existential anxieties surrounding aging and the potential loss of control over one’s life. Dementia and AD are often communicated through the imaginary of the fourth age that embodies the fears of frailty and vulnerability central to the process of othering old age. As Gilleard and Higgs (2010, p. 122) indicated, the fourth age is a “kind of terminal destination—a location stripped of the social and cultural capital that is most valued and which allows for the articulation of choice autonomy, self-expression, and pleasure in later life.” As pointed out by previous research, AD has become a visible rhetorical framing device used in debates regarding euthanasia (Johnstone, 2013). For many of the participants, dementia served as a symbolic reference point distinguishing between good old age and a health condition that is essentially dehumanizing.

Simultaneously, we can identify the existence of a lay conceptualization of dementia/AD constructed by older adults that challenges the dehumanizing representation. Older adults, in these cases, emphasized the need to understand the behavior of people experiencing dementia in the context of the altered mode of perception caused by dementia as a specific health condition. Although such representation appeared only in rare cases and was articulated exclusively in the interviews with caregivers, they represented a meaningful source of understanding dementia that enabled them to articulate the challenging feelings experienced when providing care while simultaneously reconstructing a sense of agency of the person living with dementia. What is particularly important about these lay conceptualizations of dementia is the conscious effort the participants stressed while describing their perception of dementia. The participants articulating such a conceptualization of dementia indicates that they learned to interpret and frame dementia this way through the support of activities in which they were included as caregivers or through their previous professional engagement. The emphasis on the fact that they have actively adopted this understanding of dementia highlights the critical role of such support because the conceptualization of *dementia as a specific way of approaching the world* provided an important coping mechanism for carers.

The reference to the suffering of other family members represented one of the first associations of dementia/AD articulated in the interviews. The thriving aspects of dementia were communicated primarily through the tragic impact on those surrounding people living with dementia. The effect of dementia on the mental and physical health of carers is addressed as a crucial part of the challenges associated with dementia (Farina et al., 2017). As suggested by Hall and Sikes (2017, p. 1212), “it is important to acknowledge that there are two primary ‘groups’ living with dementia” and address the impact of dementia on family members. The emphasis on the challenging impact of the illness on the family also resonates in media discourses (Van Gorp & Vercruysse, 2012). Sontag (1978, p. 6), in this context, indicated that “any disease that is treated as a mystery and acutely enough feared will be felt to be moral, if not literally, contagious.” In the case of dementia/AD, this effect manifests through the portrayal of bodies as permeable, blurring the boundaries of individual bodies and creating a representation of dementia as a condition that trespasses and affects not only the individual but also those in their proximity. Although we acknowledge the need to bring attention to the needs and experiences of family members, our research indicates that older adults’ articulations of meanings of dementia are dominated by the emphasis on the effect of such condition on family members, inadvertently making the experiences of those living with dementia less visible or even reducing them to the negative impact they have on others.

## Data Availability

The participants of this study did not give written consent for their data to be shared publicly or for the purpose of any other scientific project. Due to the sensitive nature of the research, supporting data are not available.

## Author Note

1. While dementia is a general term used for a range of symptoms, such as a decline in mental ability, memory, and problem-solving, Alzheimer’s disease is a specific degenerative brain disease marked by the symptoms of dementia. Although these terms are not the same in medical sense, in this study, the participants used the terms more as synonyms and interchangeably in their narratives. Therefore, in this paper, we only distinguish between these terms when relevant.
